# Trade-offs between survival, longevity, and reproduction, and variation of survival tolerance in Mediterranean Bemisia tabaci after temperature stress

**DOI:** 10.1093/jis/14.1.124

**Published:** 2014-09-01

**Authors:** Zhi-Chuang Lü, Yan-Min Wang, Shao-Guang Zhu, Hao Yu, Jian-Ying Guo, Fang-Hao Wan

**Affiliations:** 1 State Key Laboratory for Biology of Plant Diseases and Insect Pests, Institute of Plant Protection, Chinese Academy of Agricultural Science, Beijing 100081, China; 2 Center for Management of Invasive Alien Species, Ministry of Agriculture, Beijing 100081, China; 3 Department of Entomology, Henan Institute of Science and Technology, Xinxiang 453003, Henan Province, China

**Keywords:** heat-shock selection, heat tolerance, survival variation

## Abstract

The invasive Mediterranean *Bemisia tabaci* (Gennadius) (Hemiptera: Aleyrodidae) has emerged as one of the most common agricultural pests in the world. In the present study, we examined the cross-tolerance, fitness costs, and benefits of thermal tolerance and the variation in the responses of life history traits after heat-shock selection. The results showed that survival and longevity of Mediterranean *B. tabaci* were decreased significantly after direct or cross temperature stress and that the number of eggs per female was not reduced significantly. Furthermore, heat-shock selection dramatically increased the survival of Mediterranean *B. tabaci* within two generations, and it did not significantly affect the egg number per female within five generations. These results indicated that there was a trade-off between survival, longevity, and reproduction in Mediterranean *B. tabaci* after temperature stress. The improvement in reproduction was costly in terms of decreased survival and longevity, and there was a fitness consequence to temperature stress. In addition, heat tolerance in Mediterranean *B. tabaci* increased substantially after selection by heat shock, indicating a considerable variation for survival tolerance in this species. This information could help us better understand the thermal biology of Mediterranean *B. tabaci* within the context of climate change.

## Introduction

Ambient temperature influences virtually all biochemical and physiological processes in ectothermic animals, and it is therefore one of the most important environmental factors in determining their survival and dispersal (Cossins and Bowler 1987). Over long periods, poikilothermic insects have evolved a range of behavioral, physiological, and biochemical adaptations to withstand exposure to thermal fluctuations by phenotypic plasticity ( Kelly et al. 2012 ). Temperature variability may also be associated with marked unpredictability ( Vasseur and Yodzis 2004 , Chown and Terblanche 2007 ). Therefore, the ability of temperature fluctuations to affect activity and survival on short-term time scales is of critical importance. Although many studies of extreme temperature tolerance examined the benefits accrued ( Chown and Terblanche 2007 , Whitman and Agrawal 2009 ), far fewer studies considered the fitness costs that arise from thermal stress ( Hoffmann 1995 , Thomson et al. 2001 , Basson et al. 2012 ). Another critical problem, which is also poorly understood, is the potential for variant responses in life history traits to thermal selection.

Invasive species usually have great potential to adapt to new environments, which is the premise for a successful invasion ( Lee 2002 ). The invasive whitefly *Bemisia tabaci* (Gennadius) (Hemiptera: Aleyrodidae) has emerged in recent years as one of the most common agricultural pests in the world. Previous studies suggested that the ability of *B. tabaci* to resist heat may be one of the mechanisms that potentially underlies its invasive traits ( Cui et al. 2008 , Wan et al. 2009 , Yu et al. 2012 ). *Bemisia tabaci* has been recognized as a complex of 11 well-defined high-level groups containing at least 28 morphologically indistinguishable species ( Dinsdale et al. 2010 , De Barro et al. 2011 , Hu et al. 2011 ). One of the most widespread species is the Mediterranean *B. tabaci* ( De Barro et al. 2011 ). Field investigations have indicated that Mediterranean *B. tabaci* is becoming the predominant species in China ( Xu et al. 2006 , Hu et al. 2011 ). Our recent study reported that this species has high tolerance to short-term single temperature stress ( Yu et al. 2012 ). It is clear that in nature, animals are exposed to thermal stress by fluctuating thermal regimes ( Sinclair 2001 ). Understanding the consequences of these stresses on Mediterranean *B. tabaci* is essential for interpreting its invasive potential in the natural world ( Chown and Terblanche 2007 , Helmuth 2009 ).

To assess the relative fitness costs and benefits and the selection responses after temperature stress, the following scenarios were explored. First, we examined the tolerance ability after direct low-temperature and high-temperature stress. Second, we examined the cross-tolerance ability to temperature stress, including responses to low temperature after high-temperature pre-treatment and responses to high temperature after low-temperature pre-treatment. Third, we quantified the variant responses in life history traits in response to heat-shock selection regimes. This information could contribute to the understanding of the thermal biology of Mediterranean *B. tabaci* within the context of global climate change.

## Materials and Methods

### Insects and host plants

A stock culture of Mediterranean *B. tabaci* was established from approximately 100 adults and 500 red-eyed nymphs donated by Professor Shusheng Liu (Zhejinag University) in 2006. In our laboratory, Mediterranean *B. tabaci* was reared on tomato plants, *Lycopersicon esculentum* Mill. (Solanales: Solanaceae), in a greenhouse at 22-28°C, with 50-60% relative humidity and a photoperiod of 14:10 (L:D). The plants were grown individually in 9 cm diameter pots under the same conditions as the whitefly. The Mediterranean *B. tabaci* stock was maintained in our greenhouse for approximately four years (more than 40 generations) before the experiments started.

### Single-generation experiments

The temperature tolerance test was conducted by using a method described previously ( Cui et al. 2008 ). Bowler and Terblanche (2008) observed that adults of different ages responded differently to temperature exposure; thus, we standardized the ages of the whiteflies by using only newly emerged whitefly adults that were less than 3 h old. The temperature treatments and times were as follows: (i) -12°C for 1 h; (ii) 45°C for 1 h; (iii) 39°C for 1 h, followed by recovery at 26°C for 1 h and exposure to -12°C for 1 h; and (iv) 10°C for 1 h, followed by recovery at 26°C for 1 h and exposure to 45°C for 1 h.

For these treatments, the whiteflies were placed in a PCR or microcentrifuge tube and transferred to a low-temperature bath (DC-3015, Chi Biotechnology Co., Ltd., Ningbo, China) or to a climatic chamber (MHT350, Sanyo Electric Co., Ltd., Osaka, 85 Japan) with 60 ± 1% RH for 1 h. According to unpublished data, the maximum temperature in Xinjiang is above 45°C in the summer for 1 h during the day. Xinjiang is one of the regions in China where Mediterranean *B. tabaci* is invading ( Hu et al. 2011 ). Exposure to -12°C for 1 h was sufficient to cause changes in the whiteflies ( Yu et al. 2012 ). Furthermore, 39°C and 10°C were shown to be mild stress temperatures that can cause some physiological changes. Therefore, the temperatures and duration were selected accordingly in the present study. Adults maintained at 26°C were used as untreated controls. At least five replicates were used for each treatment.

#### Survival observation.

At least 50 newly emerged adults were collected in a 1.5 mL microcentrifuge tube. After the temperature treatment, the whiteflies were kept at 26°C for 1 h to allow them to recover. The number of whiteflies that recovered was recorded. The adults were considered dead if no appendages moved after being touched with a brush. The adults treated at -12°C or 45°C for 1 h were used as treated controls. Each replicate was one 1.5 mL microcentrifuge tube that included at least 50 adults.

#### Longevity observation.

Adults were collected and sexed under a stereomicroscope and then separated into female and male groups. A single adult was placed in a 0.2 mL PCR tube and was subjected to a temperature treatment. The surviving adults for each sex were further examined in environment chambers at a constant temperature of 26°C, with 60% RH and a photoperiod of 14:10 (L:D). Each treatment group of whiteflies (adult females and adult males) was confined on the lower leaf surface of a tomato plant by a leaf clip-on cage ( Zang et al. 2005 ). Each clip-on cage held 10 whiteflies of the same sex. Survival was checked every 24 h until all the whiteflies were dead. Each replicate was one clip-on cage including 10 whiteflies of the same sex.

#### Reproduction observation.

Adults were sexed under a stereomicroscope and then separated into female and male groups. A single adult was placed in a 0.2 mL PCR tube and was subjected to a temperature treatment. Reproduction of the surviving adults was then examined in a chamber at a constant temperature of 26°C, with 60% RH and a photoperiod of 14:10 (L:D). Each pair of whiteflies (adult female and male) was confined to the lower leaf surface of a tomato plant in a leaf clip-on cage ( Zang et al. 2005 ). Live whiteflies inside the clip-on cages were transferred to a new leaf every 48 h. The leaves with whitefly eggs were cut from the plant after the whiteflies were transferred to a new leaf. The whitefly eggs were counted under a stereomicroscope. The process was continued until the female died. If the male died during the experiment, a new male was added. Females that died within 24 h or produced no eggs were excluded from the analysis. Each replicate was one clip-on cage including one pair of whiteflies.

### Multiple-generation experiments

Adults were collected and treated at 44°C for 1 h in a climatic chamber. The whiteflies reared at room temperature (26°C) were considered the founder population. The surviving adults of the founder population were transferred to 8-10 tomato plants after heat-shock treatment, and the females laid eggs on the plants for 3 d. Then, the offspring of the first to fifth filial generations (F1 to F5) was successively generated under this treatment. Adults maintained at 26°C were used as untreated controls. At least five replicates were used for each treatment. The methods were the same as described above for “single-generation experiments.”

#### Survival observation.

Adults from each filial generation (F1 to F5) were treated. Each replicate was one 1.5 mL microcentrifuge tube that included at least 50 adults.

#### Egg number observation.

After the heat-shock treatment, each pair of whiteflies was confined to the lower leaf surface of a tomato plant in a leaf clip-on cage ( Zang et al. 2005 ), allowed to lay eggs for 48 h, and then transferred to a new leaf. The leaves with eggs were cut from the plant and eggs were counted under a stereomicroscope. The process was continued until the female died. A new male was added if the previous one died during the experiment. Females that died within 24 h or produced no eggs were excluded from the analysis. The treated population was from F1 to F5. Each replicate was one clip-on cage including one pair of whiteflies.

#### Offspring survival and female ratio.

After the heat-shock treatment, five pairs of whiteflies were confined in a leaf clip-on cage ( Zang et al. 2005 ), allowed to lay eggs for 3 d, and then were removed. The whitefly eggs were counted under a stereomicroscope. The plants with eggs were transferred to a climatic chamber until the adults emerged. The emerging adults and ratio of females were recorded. Each replicate was one clip-on cage including five pairs of whiteflies.

#### Longevity observation.

A single female or male adult was placed in a 0.2 mL PCR tube and was heat-shock treated. The surviving adults of both sexes were further examined, and five whiteflies of the same sex were confined in a leaf clip-on cage ( Zang et al. 2005 ) in a climatic chamber. Survival was checked every 24 h until all the whiteflies were dead. Each replicate was one clip-on cage including five pairs of whiteflies.

### Statistical analysis

Statistical analyses were conducted by using the SPSS package version 13 (IBM, Boulder, CO, USA). Prior to all statistical analyses, the data were examined for assumptions of normality by using the Kolmogorov-Smirnov test. Data on survival rate and female ratio were log transformed. Comparisons of survival, longevity, and reproduction in single-generation experiments were made with an independent *t* -test. For multiple-generation experiments, the survival rate, egg number, offspring survival, female ratio, and longevity of adults in F _1_ -F _5_ were analyzed by one-way analysis of variance (ANOVA) followed by Fisher’s least significant difference (LSD) test, for which the data were regarded as dependent variables and the generations were regarded as factors. The results were expressed as the means ± standard errors (mean ± SEM). The differences were considered significant when the *P* -values were < 0.05.

## Results

### Single-generation experiments

**Figure 1.**
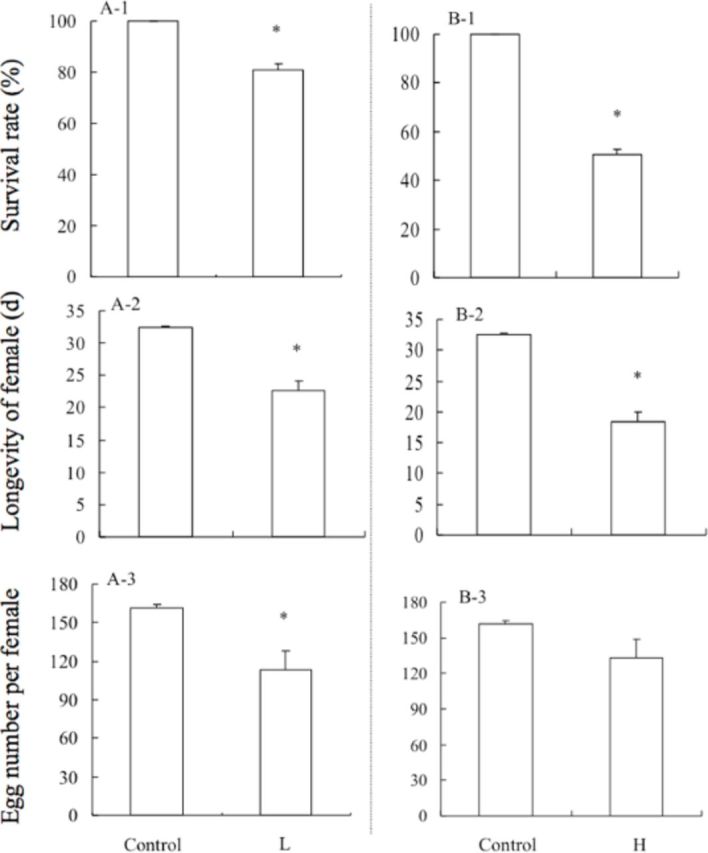
Compared with the control, the effects on survival rate, longevity, and egg number (per female) of Mediterranean *B. tabaci* adults after direct low-temperature (L) or direct high-temperature (H) exposure. Control: 26°C for 1 h; L: -12°C for 1 h; H: 45°C for 1 h. Values are the means + SEM. Asterisks denote significant differences at *P* < 0.05.

Compared with controls maintained at 26°C, the survival rate, longevity, and number of eggs per female decreased significantly after adults were exposed to -12°C for 1 h (survival rate: *t* = 12.28, *P* < 0.001; longevity: *t* = 9.72, *P* < 0.001; egg number: *t* = 4.18, *P* < 0.001) ( Fig. 1A ). There was also a significant reduction in survival rate and longevity after adults were exposed to 45°C for 1 h (survival rate: *t* = 24.71, *P* < 0.001; longevity: *t* = 15.83, *P* < 0.001), and the number of eggs per female was not greatly affected ( Fig. 1B ).

**Figure 2.**
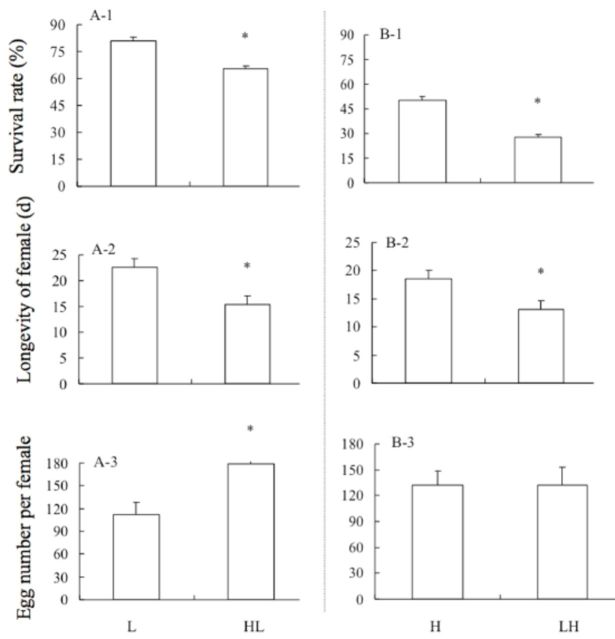
Compared with the treated control (L or H), the effects on survival rate, longevity, and egg number (per female) of Mediterranean *B. tabaci* adults after cross stress treatment (HL or LH). L: -12°C for 1 h; HL: 39°C for 1 h **—>** recovery at 26°C for 1 h **—>** -12°C for 1 h; H: 45°C for 1 h; LH: 10°C for 1 h **—>** recovery at 26°C for 1 h **—>** 45°C for 1 h. Values are the means + SEM. Asterisks denote significant differences at *P* < 0.05.

Compared with the adults exposed to -12°C for 1 h, Mediterranean *B. tabaci* adults had a significantly reduced survival rate and longevity after exposure to 39°C for 1 h followed by recovery at 26°C for 1 h and exposure to -12°C for 1 h (survival rate: *t* = 5.65, *P* < 0.001; longevity: *t* = -2.97, *P* < 0.01), and the number of eggs per female was significantly increased ( *t* = 3.16; *P* < 0.01) ( Fig. 2A ). Compared with the whiteflies exposed to 45°C for 1 h, whiteflies had a significantly reduced survival rate and longevity after exposure to 10°C for 1 h, followed by recovery at 26°C for 1 h and exposure to 45°C for 1 h (survival rate: *t* = 7.63, *P* < 0.001; longevity: *t* = 2.40, *P* < 0.05), and the number of eggs per female was not changed significantly ( Fig. 2B ).

### Multiple-generation experiments

**Figure 3.**
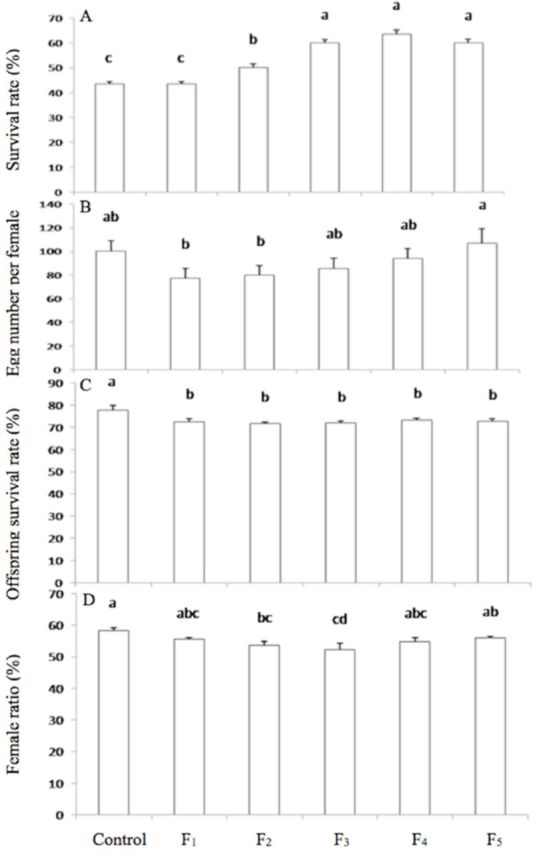
The effects on survival rate, egg number (per female), offspring survival, and female ratio of different generations of Mediterranean *B. tabaci* adults after exposure to 44°C for 1 h. Values are the means + SEM. Means marked with different letters are significantly different at *P* < 0.05.

Exposure to 44°C for 1 h had a significant effect on the survival rate of adults in different generations of Mediterranean *B. tabaci* ( *F*_5,__156_ = 43.84; *P* < 0.001). The survival rate was significantly increased after F _2_ ( *P* < 0.01) and reached a peak at F _4_ . The survival rates were 43.4, 50.2, 60.0, 64.0, and 60.1% for F _1_ , F _2_ , F _3_ , F _4_ , and F _5_ , respectively, and the survival rate of controls was 43.4% ( Fig. 3A ).

There was no significant effect on egg production in different generations of female adults when they were exposed to 44°C for 1 h ( *F*_5, 148_ = 1.59; *P* = 0.17) ( Fig. 3B ). Exposure to 44°C for 1 h had a significant effect on the offspring survival rate of different generations ( *F*_5,__129_ = 2.56; *P* < 0.05). The offspring adult survival rate was significantly reduced when compared with the 26°C control group, and there was no significant difference among different generations ( Fig. 3C ).

The female ratio of different generations of Mediterranean *B. tabaci* was significantly affected after exposure of the adults to 44°C for 1 h ( *F*_5,__18_ = 2.84; *P* < 0.05). The female ratio was significantly reduced from F _2_ to F _4_ , and the female ratios of F _1_ and F _5_ were not significantly different from that of the control ( Fig. 3D ).

**Figure 4.**
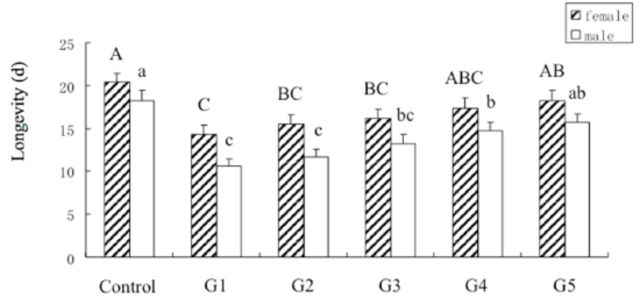
The effects on the longevity of females (striped) and males (white) of different generations of Mediterranean *B. tabaci* after exposure to 44°C for 1 h. Values are the means + SEM. Means marked with different lowercase or uppercase letters are significantly different at *P* < 0.05.

Exposure to 44°C for 1 h had a significant effect on longevity among different generations of female and male adults (female: *F*_5,__220_ = 4.55, *P* < 0.01; male: *F*_5,153_ = 7.78, *P* < 0.001). When compared with the control, the longevity of females and males was significantly decreased from F _1_ to F _3_ and from F _1_ to F _4_ ( *P* < 0.05), respectively ( Fig. 4 ).

## Discussion

In this study, we found that, when compared with control adults exposed to -12°C or 45°C for 1 h, the survival rate of Mediterranean *B. tabaci* adults was significantly decreased after exposure to 39°C for 1 h that was followed by recovery at 26°C and exposure to -12°C for 1 h, or after exposure to 10°C for 1 h that was followed by recovery at 26°C for 1 h and exposure to 45°C for 1 h, respectively. Our results are partially consistent with those of other studies. Heat and cold pre-treatment had a negative effect on cold and heat tolerance, respectively, of *Drosophila melanogaster* Meigen (Diptera: Drosophilidae) ( Sejerkilde et al. 2003 , Bubliy and Loeschcke 2005 ). Huang et al. (2007) found that a pre-treatment at 10°C did not increase survival of *Liriomyza huidobrensis* Blanchard (Diptera: Agromyzidae) in heat conditions, and that a pre-treatment at 32°C or 35°C for 4 h did not enhance the survival in cold conditions. On the other hand, Chen et al. (1987) found that a 2 h pre-treatment at 36°C could enhance the survival of *Sarcophaga crassipalpis* Macquart (Diptera: Sarcophagidae) at -10°C. Reactions to pre-treatment might have multiple physiological bases and different underlying mechanisms in different species ( Bubliy and Loeschcke 2005 ) and may include responses by stress proteins, such as heat-shock proteins ( Lü and Wan 2008 , 2011), cryoprotectants, etc. The exact reason for different levels of cold tolerance after heat exposure pre-treatment was not clear and needed further investigation to reveal the mechanism operating in Mediterranean *B. tabaci.*

It is known that temperature stress is one of main factors affecting insect fecundity. Generally, a negative correlation has been found between fecundity and increased temperature ( Irwin and Lee, Jr 2000 ; Hercus et al. 2003 ; Wiliams et al. 2003 ; Bonato et al. 2007 ; Huang et al. 2007 ; Elbaz et al. 2011 ). Huang et al. (2007) found that *L. huidobrensis* produced remarkably fewer eggs following a 4 h exposure to 10, 32, or 35°C. Interestingly, in the current study, we found that the number of eggs per female was not reduced significantly after exposure of adults to 45°C for 1 h or after exposure to 10°C for 1 h followed by recovery at 26°C for 1 h and exposure to 45°C for 1 h. There might be a few possible reasons for this phenomenon. The first one is that this exposure to heat with or without a cold pre-treatment might not produce negative effects on the reproductive system of Mediterranean *B. tabaci.* The second possibility is that the interaction of exposure temperature and time was essential to cause a change in the reproductive system. An exposure to 42°C for 1.5 h was shown to have a negative effect on the fecundity of Mediterranean *B. tabaci* ( Elbaz et al. 2011 ), but the number of eggs was not affected after exposure of adults to 45°C for 1 h in the present study. These results indicate that regulation in the reproductive system of Mediterranean *B. tabaci* is complex under temperature stress conditions, and the underlying mechanism is not clear and needs further experimentation to be identified. The third possibility is that there was a trade-off between survival and reproduction in the whiteflies after temperature stress. Marshall and Sinclair (2010) suggested that a trade-off between immediate survival and future fitness could result from multiple stresses. They reported that multiply exposed *D. melanogaster* had a higher survival rate than flies exposed to cold for the same amount of time without pre-treatment, and that the survival improvement was costly, resulting in a lower intrinsic rate of population increase. We therefore suggest that the improvement in reproduction observed in Mediterranean *B. tabaci* might have been costly in terms of decreased survival and could be a fitness consequence to temperature stress.

Insect lifespan is correlated to environmental temperature. Bonato et al. (2007) reported that the lifespan of Mediterranean *B. tabaci* adults was negatively correlated with increased temperature, similar to the results of this study. Smith (1958) first reported that a short period of heat exposure prolonged the lifespan of female *Drosophila subobscura* Collin (Diptera: Drosophilidae). Bourg et al. (2001) found that young adult *D. melanogaster* exposed to a daily, 5 min, 37°C heat shock for 5 d experienced increased longevity within 2 d, but that longer shock times ( **>** 10 min) had a negative or neutral effect on longevity. Hercus et al. (2003) showed that repeated heat exposure can extend the lifespan of *D. melanogaster* females. These results indicate that insect longevity is very sensitive to different temperature changes. In the present study, we found that direct or cross temperature stress significantly reduced the longevity of Mediterranean *B. tabaci* adults and that egg laying was not significantly reduced. It is possible that there was a trade-off between lifespan and reproduction in Mediterranean *B. tabaci* under stress conditions, although the underlying mechanism is not clear and requires further study.

Furthermore, our present results showed that heat-shock selection dramatically increased survival of Mediterranean *B. tabaci* within two generations, which indicated that there was sufficient genetic variation to permit a rapid increase in survival. Studies have reported that selection significantly increased knockdown resistance of *D. melanogaster* with increasing knockdown temperature ( Gilchrist and Huey 1999 ) and increasing knockdown time ( McColl et al. 1996 , Bubli et al. 1998 ). Sambucetti et al. (2010) found that lines of *Drosophila buzzatii* Patterson & Wheeler (Diptera: Drosophilidae) significantly diverged for increased heat thermotolerance after 12 generations of artificial selection. On the other hand, Kelly et al. (2012) reviewed that several investigations examining acclimation to thermal environments had concluded that phenotypic plasticity did not always lead to increased fitness in an altered environment or even might be maladaptive. The present results showed that the number of eggs produced was not significantly affected within five generations of selection, which suggested that there was less variation in egg production and that phenotypic plasticity after selection could not increase this fitness parameter. Maybe, there was a trade-off between survival and reproduction in Mediterranean *B. tabaci* after the thermal stress selection experiment.

Collectively, the present results showed that the survival and the longevity of Mediterranean *B. tabaci* were significantly decreased after direct or cross temperature stress and that the number of eggs per female was not significantly reduced. Furthermore, heat-shock selection dramatically increased the survival of Mediterranean *B. tabaci* within two generations, and the number of eggs per female was not significantly affected within five generations of selection. These results indicated that there was a trade-off between survival, longevity, and reproduction in Mediterranean *B. tabaci* after temperature stress. The maintained level of reproduction was costly in terms of decreased survival and longevity, and there was a fitness consequence to temperature stress. In addition, heat tolerance to high temperature was expressed by selection for increased survival, indicating a considerable variation in responsiveness to thermal stress. The present study examined only the biological traits of Mediterranean *B. tabaci* after direct or cross temperature stress or thermal stress selection, and the underlying mechanisms of these phenomena remain unknown. In future work, we will consider the molecular mechanisms (such as heat-shock protein expression) of thermotolerance after different stress treatments.
